# Distribution of spine classes shows intra-neuronal dendritic heterogeneity in mouse cortex

**DOI:** 10.1117/1.NPh.12.1.015001

**Published:** 2024-12-19

**Authors:** Carina C. Theobald, Ahmadali Lotfinia, Jan A. Knobloch, Yasser Medlej, David R. Stevens, Marcel A. Lauterbach

**Affiliations:** Saarland University, Molecular Imaging, Center for Integrative Physiology and Molecular Medicine, Homburg, Germany

**Keywords:** dendritic spine, spine morphology, spine shape, dendrite-specific, super-resolution stimulated emission depletion microscopy, three-dimensional analysis

## Abstract

**Significance:**

Neuronal dendritic spines are central elements for memory and learning. Their morphology correlates with synaptic strength and is a proxy for function. Classic light microscopy cannot resolve spine morphology well, and techniques with higher resolution (electron microscopy and super-resolution light microscopy) typically do not provide spine data in large fields of view, e.g., along entire dendrites. Therefore, it remains unclear if spine types are organized on mesoscopic scales, despite their undisputed importance for understanding the brain.

**Aim:**

Recently, it was shown that the distribution of spine type is dendrite-specific in the turtle cortex, suggesting a mesoscopic organization, but leaving the question open if such a dendrite specificity also exists in mammals. Here, we determine if such a difference in spine-type distribution among dendrites also exists in the mouse brain.

**Approach:**

We used super-resolution stimulated emission depletion microscopy of complete dendrites and advanced morphological analysis in three dimensions to decipher morphological differences of spines on different dendrites.

**Results:**

We found that spines of different shapes decorate different dendrites of the same neuron to a varying extent. Significant differences among the dendrites are apparent, based on spine classes as well as based on quantitative descriptors, such as spine length or head size.

**Conclusions:**

Our findings may indicate that it is an evolutionarily conserved principle that individual dendrites have distinct distributions of spine types hinting at individual roles.

## 1 Introduction

Most excitatory synapses in the vertebrate brain are found on dendritic spines.^1^ These spines are believed to regulate synaptic strength.^2^ The spine neck serves for diffusional and electrical isolation of the synapse from the dendritic shaft.^3^^–^^5^ Changes in spine structure are relevant for memory, cognition,^6^ and mental disorders,^7^ e.g., the size and shape of the spines are correlated with the strength of postsynaptic currents.^8^

The links that have been reported between morphological characteristics and function of spines^2^^,^^9^^,^^10^ propose that spine morphology can be a valuable proxy for function, e.g., synaptic strength and learning rules may depend on morphology.^10^

To decipher the role of spine shape, highly resolved morphological data are desirable but are rarely available. Typically, spines are sorted into a few groups, but these categories are likely the result of binning a continuum.^11^^,^^12^ In addition, the class assignment depends on the resolution with which they are imaged.^13^^,^^14^ Super-resolution images render, e.g., many fewer “stubby” spines, and provide a much more detailed visualization than do two-photon microscopy images, which are frequently used for spine imaging and classification.

Classical light microscopy allows for imaging large volumes but lacks the resolution needed to resolve the intricate details of spine morphology. Therefore, serial-section electron microscopy is often considered the gold standard for creating three-dimensional (3D) morphological reconstructions of individual spines and dendrites.^3^^,^^15^ However, with this technique, the reconstructions seldom contain complete dendrites or neurons, with some exceptions.^16^^,^^17^

A recent study employed a combination of lattice light-sheet microscopy and expansion microscopy to conduct high-resolution large-scale imaging of mouse brains.^18^ This study found spine characteristics to be specific to different layers of the brain. Other studies, which relied on widefield or confocal microscopy with limited spatial resolution, have reported differences between spines on apical and basal dendrites in human pyramidal cells.^19^^,^^20^ However, we are not aware of similar descriptions of such differences in mice.^21^

Super-resolution stimulated emission depletion (STED) microscopy^22^^–^^24^ offers the advantages of time-resolved high-resolution imaging^25^^–^^28^ and the ability to discern sub-cellular structures with excellent contrast.^29^^,^^30^ Importantly, it can provide high spatial resolution while allowing imaging of large fields of view. Single dendrites can be imaged, omitting empty space between them, tremendously reducing the total volume imaged without losing structural information.^31^

With this strategy, we have recently shown that spines of different morphological classes are not randomly distributed on the dendrites of one and the same neuron but that some dendrites carry preferably spines of one class, whereas others are preferentially decorated with spines of other classes.^31^ Thus, neuronal dendrites possess a distinct set of spines that distinguishes them from other dendrites of the same cell. Such a mesoscopic organization might have a profound impact on the computational organization and capacity of neurons. However, this dendrite-dependent organization of spines has only been demonstrated so far in the red-eared slider (*Trachemys scripta elegans*), i.e., in a reptilian brain.

Here, we used the super-resolution capabilities of STED microscopy to image in 3D complete dendrites of spiny neurons in the mouse cortex. We examined systematically the variability of spines on individual dendrites with high-resolution nanometer-scale morphological analysis to investigate whether a similar distinction of dendrites exists in the cortex of mammalian brains. Our investigation showed variations in spine-type composition among the dendrites of individual neurons. These findings suggest that the individuality of dendrites exists not only in reptiles but also in mammals.

## 2 Results

**Fig. 1:**
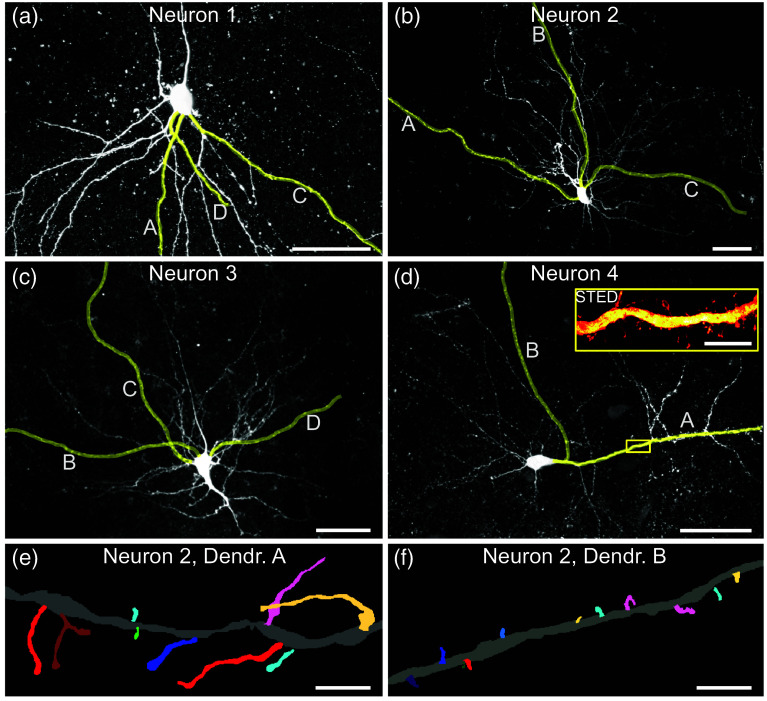
Cortical neurons. (a)–(d) Confocal overview images of the perisomatic region of the neurons filled with markers in the mouse cortex (maximum-intensity projections). Letters and yellow markings identify the individual dendrites, which were then imaged with STED microscopy [inset in panel (d)], eventually also beyond the field of view shown here. Individual neurons were filled with biocytin and revealed with the streptavidin-coupled fluorophore Atto 647N. Scale bars, 50  μm and 5  μm in the inset. (e) and (f) Segmentation of individual spines from two dendrites of the same neuron highlights differences (maximum-intensity projections shown). Scale bars, 2  μm.

We imaged with high-resolution STED microscopy nearly all spines of the selected dendrites of neurons in the mouse cortex. Individual neurons were marked by filling them with markers via patch pipettes (see Sec. 4). We concentrated on three neurons from the same mouse, excluding the effects of inter-animal variability [Figs. 1(a)–1(c)]. Three dendrites of each neuron were imaged. To confirm that the observed differences in our experiments were not specific to this one animal, we imaged two dendrites of one neuron in another animal [Fig. 1(d)], where we again found differences among dendrites. In total, 2171 dendritic spines were imaged, of which 1765 spines were well enough resolved to be segmented to determine their morphology with high resolution and to analyze their shape. The findings were then further corroborated, independently of patching and slicing, in dendrites expressing the fluorescent marker protein tdTomato.

The initial overview 3D stacks of the neurons were acquired using low-magnification confocal microscopy (Fig. 1). Subsequently, individual dendrites were piecewise imaged in 3D at high resolution with STED microscopy [Fig. 1(d), inset]. A key advantage of our experimental approach was the isolated labeling of cells, ensuring high contrast. This allowed us to limit imaging solely to the dendrites. As a result, the total imaged volume was significantly reduced, ranging from 3.5% to 19.5% of the cuboid enclosing the dendrites imaged. This strategy not only minimized bleaching and acquisition time but also reduced the amount of data requiring storage and handling.

Manual segmentation of 1765 imaged spines enabled us to analyze their morphology in intricate detail; visually, differences are discernible among the spines on some dendrites [Figs. 1(e) and 1(f)]. Based on their morphology and length in 3D, the spines were then clustered. Independently from the clustering, they were characterized with 10 morphological descriptors (such as neck width, head width, length, and thickness variations).

### 2.1 Classification of the Spines

**Fig. 2:**
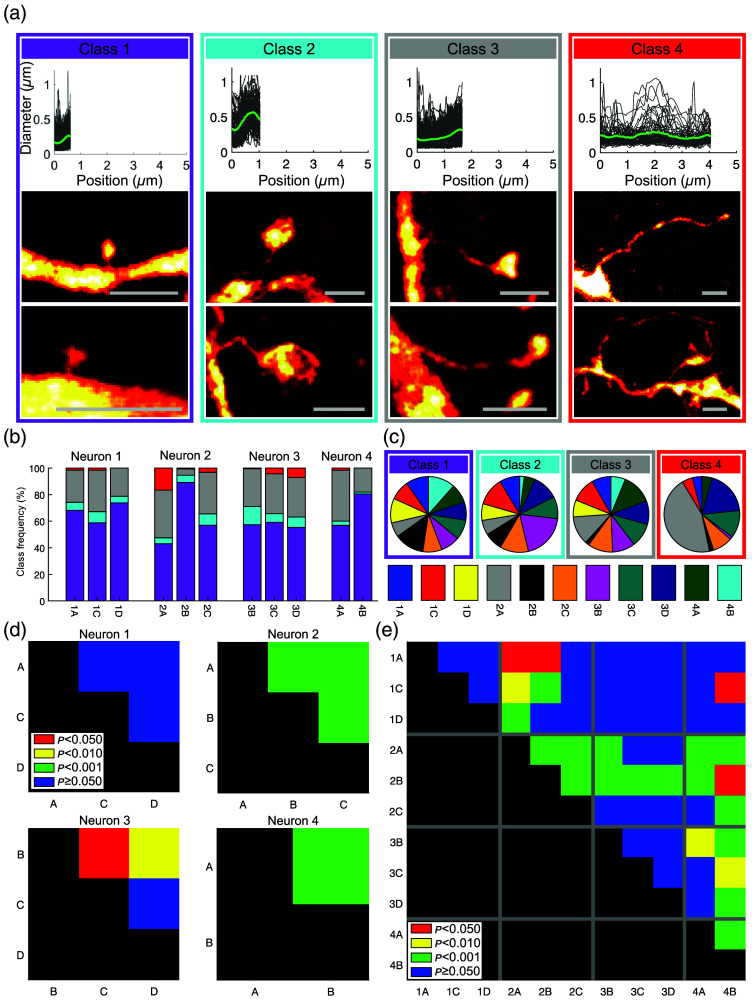
Spine classes are inhomogeneously distributed on the dendrites. (a) Spines were categorized into four classes by hierarchical clustering of shape and length: upper panels—individual diameter profiles (gray) and average profile of each class (green). The total lengths of the profiles were scaled to the average spine length of the respective class; lower panels—representative spine examples. Scale bars, 1  μm. (b) Relative abundance of each spine class on the dendrites, the distribution differs among dendrites. Numbers 1 to 4 refer to the four neurons, letters to the dendrites (see Fig. 1). Color code as in panel (a). (c) Pie charts showing which fraction of a specific spine class is found on each dendrite. (d) Pairwise Pearson’s chi-square tests confirm significant differences among the repartition of the classes on the dendrites within three of the four neurons. p values color coded: p≤0.05 red, p≤0.01 yellow, p≤0.001 green, and p>0.05 blue. All tests have been corrected for multiple comparisons. (e) Pairwise Pearson’s chi-square tests for all dendrites of all cells.

Although spines are typically classified into a few categories, this should be considered as a binning of a continuum.^11^^,^^12^ Pchitskaya and Bezprozvanny found that such data are better described by clustering than by classification into predefined groups.^11^ Therefore, we employed hierarchical clustering for classifying the spines based on their length and morphology (diameter profile) [Fig. 2(a)].

Using the Davies–Bouldin criterion^32^ to determine the optimal number of clusters, we identified four classes. These classes, as defined by the unbiased outcome of the clustering, are visually similar to the classically used “stubby,” “mushroom,” “thin,” and “filopodia” classification^33^ [Fig. 2(a)]. However, in our data, even for the shortest spines, we could typically resolve a neck [Fig. 2(a)], which is not unexpected because “stubby” spines without a neck are likely an artifact of insufficient imaging resolution;^13^^,^^14^ we do not see truly “stubby,” i.e., neckless, spines. The high-resolution images revealed a large heterogeneity of spine shapes.

### 2.2 Difference in Spine-Type Distribution among Dendrites

In the next step, we tested if the spines of different classes are randomly distributed on individual dendrites or if some spine classes decorate some dendrites more often than others, as observed in reptiles.^31^

A chi-square test indicated that the spines of the different classes were overall not homogeneously distributed among the dendrites (Pearson’s chi-square test across all cells, 11 dendrites, $p<1.0\times 10^{-34}$). In addition, chi-square tests for the four individual neurons indicated that within three of the four neurons, the spine classes were not homogeneously distributed among the dendrites of one and the same neuron (for the four neurons: p>0.44, $p<2.8\times 10^{-20}$, $p<1.1\times 10^{-2}$, $p<1.4\times 10^{-5}$). Some spine classes were found significantly more often on some dendrites of the same neuron than on the others [Fig. 2(b)]. This means that dendrites had a specific signature, based on the ratios of the spine classes present on the dendrite. Examination of the distribution of spine classes on individual dendrites [Fig. 2(c)] revealed that about half of the filopodia-like spines were found on one of the eleven dendrites, dendrite 2A. When comparing the spine composition of the dendrites pairwise [Fig. 2(d)], we found significant differences among the dendrites of neuron 2 for all three comparisons. On neuron 3, dendrite B differed significantly from the two others. On neuron 4, the dendrites were significantly different; no significant differences were observed within neuron 1. The pairwise comparison of all dendrites (also across neurons) revealed many significant differences; dendrites 2A and 2B were distinct from particularly many other dendrites [Fig. 2(e)]. We did not see a clear trend along the dendrites (Fig. S1 in the Supplementary Material).

To confirm that the observed differences among dendrites are not the result of a particular labeling or sample preparation method, we repeated the analysis using transgenic animals expressing tdTomato as the marker protein in a sparse subset of neurons (Fig. S2 and Supplementary Methods in the Supplementary Material). After transcardial perfusion and fixation of the entire brain, vibratome slices were prepared and stained against tdTomato to obtain strong staining of individual neurons [Fig. S2(a) in the Supplementary Material] for STED imaging. Three entire dendrites were recorded via STED microscopy, segmented, and clustered [Fig. S2(b) in the Supplementary Material]. Again, we found significant differences in the cluster distribution among some dendrites [Figs. S2(c) and S2(d) in the Supplementary Material], corroborating that such differences are not only consistent across various sample preparation and labeling techniques but also not specific to the initially examined mice.

### 2.3 Quantitative Spine Descriptors Confirm that Dendrites Differ from Each Other in Spine-Type Distribution

**Fig. 3:**
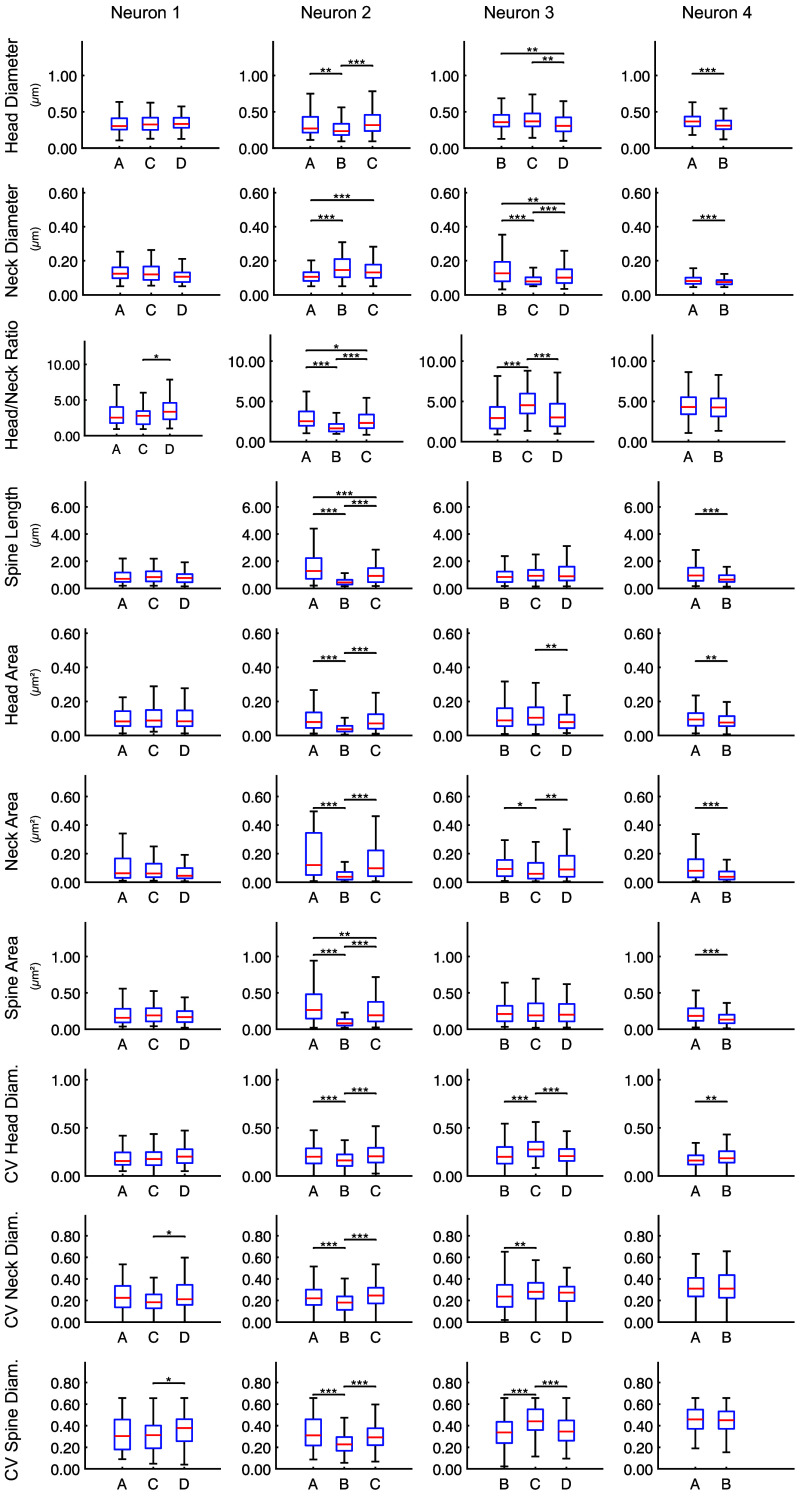
Spine descriptors are distinct among dendrites. Quantitative spine descriptors show significant differences in dendrites of the same neuron. Box plots show the median and quartiles; the whiskers extend to the most extreme data points not considered outliers (outliers not depicted). *p≤0.05, **p≤0.01, and ***p≤0.001. All tests have been corrected for multiple comparisons (Kruskal–Wallis tests). Each column represents one neuron. All tested descriptors differ significantly among several dendrites, with the fewest differences in neuron 1. Width variability within spines given as coefficient of variation (CV).

**Fig. 4:**
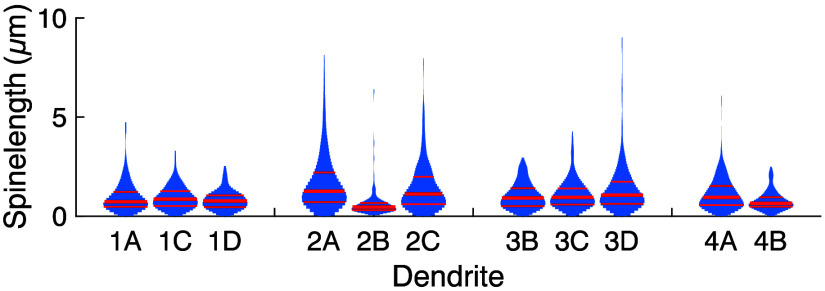
Spine length distributions vary among dendrites. The spine lengths show distinct distributions on different dendrites. Violin plots: red lines show the median and quartiles.

The inhomogeneous distribution of spine classes clearly shows that spines of a given class are present at different frequencies on individual dendrites. However, the classification of the spines based on hierarchical clustering remains somewhat abstract. We therefore tested if quantitative morphological descriptors of spines including head diameter; neck diameter; their ratio; spine length; head, neck, and spine area; and the thickness variations within individual spines are different among the dendrites (Fig. 3, see Sec. 4 for the detailed definitions of the descriptors). In all four neurons, significant variations were observed among their respective dendrites in at least some of these descriptors. Significant differences among the dendrites in terms of average spine head and neck diameters, their ratio, average head and neck areas, and the width variations within individual spine heads, necks, and entire spines were found in three of the four neurons. Spine length and spine area differed significantly among dendrites in two of the four cells. Particularly many significant differences were found in neuron 2; all analyzed descriptors showed significant differences among dendrites. Consistent with neuron 1 showing no variation in spine class composition [Fig. 2(d)], it had the fewest significant differences in quantitative spine descriptors. The pairwise dependences of some of the spine parameters and the relationship with the spine class are shown in Fig. S3 in the Supplementary Material. When randomly shuffling the spines among dendrites as a control, no significant differences were observed anymore, as expected (Fig. S4 in the Supplementary Material). We examined the spine length distributions in more detail and found distinct variations among the different dendrites (Fig. 4).

### 2.4 Spinules, Branched Spines, and Contact Sites

**Fig. 5:**
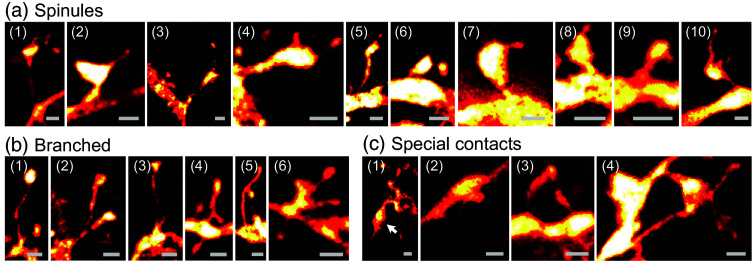
Spinules, branches, and special contacts. (a) Spines with spinules. (b) Branched spines. (c) Spines with unusual contacts. Arrow in (1) points to a synapse. Scale bars, 500 nm.

The super-resolution 3D images enabled us to resolve several spines with spinules^34^^,^^35^ of various forms on the cortical neurons [Fig. 5(a)]. Spinules are protrusions from the spine heads,^35^^,^^36^ whose role in synaptic function still remains elusive.^37^ Spinules that we found in the mouse cortex could be long and thin, eventually with a thickening at the end, resembling a tiny spine head. This looked like a tiny spine growing out of a larger spine [Figs 5(a), (1)–(6)]. In other cases, the spinules were only short protrusions from the spine head [Fig. 5a, (7)–(9)]. Sometimes, multiple spinules were observed on the same spine head [Fig. 5(a), (4) and (10)]. Comparing the distribution of spinules on the four spine classes, we found most spinules on the spines of class 3 (Fig. S5 in the Supplementary Material). Spinules were also observed on the neurons that express the fluorescent marker protein tdTomato (Fig. S6 in the Supplementary Material).

Some spines were branched [Fig. 5(b)], often with both branches bifurcating very close to the dendrite [Figs. 5(b), (1)–(4)], but also structures such as a head on the side of a filopodium were observed [Fig. 5(b), (5)]. One spine resembled a large spine with a large spinule and a thin side branch [Fig. 5(b), (6)]. Some spines were intriguing due to their contacts [Fig. 5(c)]: One structure resembled a synapse with a stained axon terminal [Fig. 5(c), (1), arrow]. As only one neuron was stained, this was likely an auto-synapse. A spine was found bending back to a thickened part of the dendrite [Fig. 5(c), (2)]. Furthermore, we observed converging spines that seemed to make contact with the same (unstained) structure [Fig. 5(c), (3)]. In one case, a “thin” spine passed by a mushroom-like spine with a branched spinule [Fig. 5(c), (4)].

## 3 Discussion and Conclusion

Morphology is the foundation for physiological function.^38^ Studying nano-scale morphology requires sparse labeling with high contrast, well-preserved samples and a microscopy technique that can provide high resolution. Here, we presented a systematic investigation of neuronal spine morphology in murine cortical samples using super-resolution STED microscopy and large-scale analysis in 3D. This analysis is in line with prior research conducted in a non-mammalian species, expanding our understanding of dendritic spine ultrastructure in mice and across species.

Recently, we found that dendritic spines are not homogeneously distributed among neuronal dendrites in the turtle cortex.^31^ Here, we show that such a dendrite dependence of spine classes in individual neurons also exists in the cerebral cortex of mice. This might indicate that individual dendrites use specialized sets of spines for computation, and this phenomenon might have been present already in the last common ancestor of reptiles and mammals. With super-resolution STED microscopy, we imaged eleven dendrites of spiny neurons in mouse cortex with 2171 dendritic spines, of which 1765 were sufficiently well resolved for detailed analysis. We analyzed almost entire dendrites, which allowed us to compare the morphology of spines among the dendrites (Fig. 1). Strikingly, we found that in the mouse cortex—as in the turtle^31^—the dendrites were often decorated by a distinct mixture of spine classes (Fig. 2) as defined by clustering of spine shape and length. Also, several quantitative measures such as head and neck diameter, length, and surface area corroborated the lack of homogeneity of spine shape among the dendrites (Fig. 3). Importantly, the spine-type (or class) composition was different among the dendrites of the same neuron in three of the four neurons analyzed. These differences, both on the level of classes and the level of several quantitative descriptors, were statistically significant. The spine composition of one dendrite (dendrite “A” of the second neuron) stood out particularly, with many filopodia-like long spines [Figs. 2(b) and 2(c), Figs. 3 and 4]. Furthermore, differences in the spines on dendrites of different cells were seen, both in the same animal and among animals. To our knowledge, differences in the spines on individual dendrites have only been reported in detail for turtles^31^ and as a crude difference for basal/apical dendrites of human pyramidal neurons.^19^

To characterize and classify spine shapes, we used hierarchical clustering rather than sorting into predefined standard classes (“stubby,” “mushroom,” “thin,” and “filopodia”) because clustering is considered preferable over classification into predefined groups.^11^ Nonetheless, our clustering results match reasonably well the standard classes.^33^ However, as expected from other super-resolution spine studies,^13^^,^^14^ we observed distinguishable necks, even in the smallest spines.

Another feature that becomes clearly visible in our data is the presence of spinules on the spines [Fig. 5(a)], i.e., protrusions from the spine head, which we identified on more than 30 spines. Typically, spinules are only reported in electron microscopy reports,^35^ with only a few studies using light microscopy;^35^^,^^36^^,^^39^ they have likely often been missed in diffraction-limited microscopy studies because of their nano-scale.^35^ Sometimes they were described as filopodia emanating from the head,^35^^,^^39^^,^^40^ whereas we can clearly show that some of them have themselves a tiny “head” (Fig. 5). Functions as diverse as material transport between pre- and postsynapse,^41^ retrograde signaling,^42^ structural anchor,^37^ and interaction with glia^42^ have been proposed.^35^ Spinules are probably involved in synapse formation and stabilization of mature spines; altered spinules might play a role in psychiatric disorders.^35^ Furthermore, it was found that glutamate receptors are particularly mobile in spinules, but overall, the physiological functions of spinules remain poorly understood,^43^ and their role in the function of synapses is elusive.^37^

Our analysis of the spines on several dendrites of the same neurons suggests that dendritic compartments defined by spine composition are a feature that is not only seen in reptiles but also in mammals. The observation that the majority of dendrites in this study exhibited a distinct set of spine types suggests a potential specialization of individual dendrites for information processing, which is in line with reports that dendrites behave electrically as semi-independent compartments.^44^ For the synaptic and electric properties of spines, their shape is critical,^2^ and neck length influences calcium dynamics.^45^ Postsynaptic potential amplitudes measured in the soma negatively correlate with the length of spine necks^2^ and calcium kinetics in spines depends on neck length.^45^^,^^46^ Mushroom spines have been attributed to sustained and strong synaptic activity,^7^ and it was suggested that large spines might be traces of long-term memory, whereas small spines are sites for induction of long-term potentiation.^8^

Functional data related to the spine composition of individual dendrites are still missing, but the morphological differences of the spines on different dendrites are very prominent and links of form to function are numerous.^2^^,^^7^^,^^8^^,^^45^^,^^46^ For pyramidal cells of rodents, it was shown that single dendrites are sensitive to the activation sequence of their synapses; the dendrites act thus as individual processing compartments.^47^ Our finding that spine composition differs among dendrites might suggest that these differences serve to tweak such dendritic computations.

## 4 Appendix: Materials and Methods

### 4.1 Animals and Tissue Preparation

This study was carried out at Saarland University in strict accordance with the recommendations of European and German guidelines for the welfare of experimental animals. The procedures involving animal husbandry and care were conducted in conformity with the institutional guidelines that are in compliance with national German and international laws and policies (DIRECTIVE 2010/63/EU; Tierschutzgesetz; Tierschutz-Versuchstier-Verordnung; FELASA guidelines). Animals were kept under a 12-h light/dark cycle with food *ad libitum*. The animals were sacrificed according to § 4 (3) Tierschutzgesetz and § 2 Tierschutz-Versuchstierverordnung or according to § 8 (animal license number 03/2021) approved by the “Landesamt für Gesundheit und Verbraucherschutz” of the state of Saarland.

C57BL/6N mice of either sex (neurons 1 to 3 female, neuron 4 male, 34 to 36 days old) were decapitated after cervical dislocation. The brains were dipped into ice-cold, oxygenated (with 5% ${\mathrm{CO}}_{2}$ and 95% $O_{2}$) artificial cerebral spinal fluid denoted as cutting solution (${\mathrm{ACSF}}_{\mathrm{CS}}$), consisting of (in mM) 87 NaCl, 3 KCl, 1.2 ${\mathrm{NaH}}_{2}{\mathrm{PO}}_{4}$, 25 ${\mathrm{NaHCO}}_{3}$, 3 ${\mathrm{MgCl}}_{2}$, 0.5 ${\mathrm{CaCl}}_{2}$, 25 glucose, and 75 sucrose, with a pH of 7.4. Acute coronal slices (300  μm thick) from the cortex were cut with a vibratome (Leica VT1200 S; Leica Biosystems, Wetzlar, Germany). They were then stored in an oxygenated incubation solution (${\mathrm{ACSF}}_{\mathrm{IS}}$), composed of (in mM) 126 NaCl, 3 KCl, 1.2 ${\mathrm{NaH}}_{2}{\mathrm{PO}}_{4}$, 25 ${\mathrm{NaHCO}}_{3}$, 2 ${\mathrm{MgCl}}_{2}$, 1 ${\mathrm{CaCl}}_{2}$, and 15 glucose on a nylon mesh slice holder. The slices recovered for 30 min at 35°C and were then maintained at room temperature with continuous oxygenation for at least 1.5 h and not longer than 6 h before patching.

### 4.2 Patching and Staining

Neurons in the cortex were marked by filling them individually with biocytin. For biocytin-filling, conventional whole-cell recordings were performed with borosilicate glass pipettes with resistances of 3 to 6 MΩ, which were connected to the head stage of an EPC-10 patch-clamp amplifier controlled by the Patchmaster software (Heka Electronic, Reutlingen, Germany). The extracellular solution contained (in mM) 12.6 NaCl, 3 KCl, 1 ${\mathrm{MgCl}}_{2}$, 2.5 ${\mathrm{CaCl}}_{2}$, 15 glucose, 1.2 ${\mathrm{NaH}}_{2}{\mathrm{PO}}_{4}$, and 25 ${\mathrm{NaHCO}}_{3}$ and was adjusted to a pH of 7.4. The pipette solution contained (in mM) 144 Cs-aspartate, 1 ${\mathrm{MgCl}}_{2}$, 2 Mg–ATP, 0.3 ${\mathrm{Na}}_{2}$–GTP, 5 Cs–EGTA, 3.5 ${\mathrm{CaCl}}_{2}$, and 10 Cs–HEPES with a pH of 7.2. Biocytin was added to the pipette solution on the day of the recording, resulting in a final osmolarity of ∼280  mOsm and 0.5% biocytin. Recordings were done at room temperature. Following giga-Ohm seal formation, the membrane patch was disrupted by manual suction. As soon as the whole cell configuration was achieved, the capacitance was corrected, and a current–voltage curve was recorded from a holding potential of −70  mV. Neurons with high membrane resistance and robust sodium current (upon depolarization) and a low access resistance (<30  MΩ) were then allowed to fill with biocytin for at least 20 min. After the removal of the pipette, the slices were fixed at 4°C overnight in 4% paraformaldehyde for staining and processing.

Slices were then washed three times in phosphate-buffered saline (PBS) (in mM: 137 NaCl, 2.7 KCl, 10 ${\mathrm{Na}}_{2}{\mathrm{HPO}}_{4}$, 1.8 ${\mathrm{KH}}_{2}{\mathrm{PO}}_{4}$, pH 7.4) and incubated for 1 h in 2% Triton X100 in PBS (Sigma-Aldrich, St. Louis, Missouri, United States) followed by an overnight incubation with Atto 647N-conjugated streptavidin (Sigma-Aldrich, 2  μg/ml in PBS). Slices were washed three times 20 min in PBS before embedding in Mowiol. Spacers (adhesive sheets, Sigma-Aldrich) matching the slice thickness were placed between the cover slip and the slide to protect the brain samples from squeezing. Care was taken that the sample was close to the cover slip; #1.5H coverslips (170±5  μm thick, Marienfeld, Lauda-Königshofen, Germany) were used.

### 4.3 STED Imaging

Neurons were imaged with an inverted STED microscope (Expert Line, Abberior Instruments, Göttingen, Germany). For high-resolution imaging, a 100× silicone oil immersion objective (UPLSAPO100XS, Olympus Germany, Hamburg, Germany) was used with a voxel size of $20\times 20\times 300\;{\mathrm{nm}}^{3}/25\times 25\times 300\;{\mathrm{nm}}^{3}$. Atto 647N was excited with a pulsed 640-nm laser, and a 775-nm laser was used for STED with the typical toroidal (“donut”) focus. The power of the STED beam was ∼390  mW at the back focal plane of the objective. The detection window was 650 to 720 nm. Linear deconvolution (Wiener filtering) in two dimensions (2D) was applied to each plane of the image stacks using custom-written routines in Matlab (The Mathworks, Natick, Massachusetts, United States). As a point spread function, a 2D Lorentzian function was used with the same full width at half maximum as the 50-nm resolution that was measured on test particles (40-nm red fluorescent beads, Abberior Instruments, Göttingen, Germany).

Dendrites were identified in overview images in confocal mode with a 30× silicone oil immersion objective (Olympus UPLSAPO30XS). Dendrites with favorable positioning for high-resolution imaging (<20  μm from the surface) were chosen for STED imaging and further analysis. They were piecewise imaged with small overlaps among consecutive STED image stacks. Individual stacks covered a depth of ∼4 to 10  μm, depending on the orientation of the dendrite. Overview images (Fig. 1) were stitched with ImageJ and saturated for display to make dendrites clearly visible.

### 4.4 Ultrastructural Analysis and Clustering

Ultrastructural morphological analysis of the spines was implemented as described in detail in Ref. 31. First, the skeletons of the spines and dendrites in the 3D datasets were drawn with webKnossos,^48^ i.e., the centerline of the spine was marked in each imaging plane that contained parts of the spine, neck and head region were manually marked. Further analysis was done with Matlab (Mathworks, Natick, Massachusetts, United States): spines were then manually segmented in the deconvolved images along the skeletons; in each imaging plane, the outline of the spine was drawn next to the skeleton. The restriction to the region around the skeleton ensured that the spine was always outlined in the plane where it appeared sharpest. This led for each spine to a skeleton with corresponding segmentation across several imaging planes. In the next step, the spine width (i.e., the width of the segmented area) was calculated automatically every ∼20  nm (interpolated where necessary) perpendicular to the skeleton (and in the imaging plane), giving the complete width profile of the spines. The analysis is thus based on width profiles in the imaging plane that follow the spine through the 3D tissue and not on projections. Due to the lower resolution along the optical axis (“z-resolution”), no attempt for a volume reconstruction was made. If the complete spine morphology was not clearly recognizable, the spine was excluded from further morphology analysis.

The quantitative descriptors (Fig. 3) are based on these width profiles and were defined as follows: “head diameter” as the largest diameter within the head region, “neck diameter” as the smallest diameter in the neck region, and “spine length” as the length along the skeleton in 3D from the attachment point of the spine on the dendrite to the end of the head. “Spine area,” “neck area,” and “head area” as the integral of the diameter values along the spine, approximated as the sum over all diameter profiles times the actual sampling intervals. These areas followed the spines in 3D and were not just calculated as 2D projections. Diameter variation within each spine was expressed as the coefficient of variation (CV) of all diameter values in each spine (respectively the neck and head regions).

For the classification of spines by their morphology, hierarchical clustering (Euclidean distances and Ward algorithm) was used based on the diameter profile of the spines and their length. To separate the diameter profile of the spines (measured every ∼20  nm) from their length, the profiles were resampled to 100 sampling points per spine. Shape served as a clustering criterion independently of the spine length. The spine length was included in the clustering as an independent feature. Dimensions 1 to 100 contained the diameter profile of the spine from dendrite to end and dimensions 101 to 175 the length (75 times repeated to balance the influence of the length in comparison to the 100 spine diameter values). To equalize the impact of all dimensions during the clustering, we rescaled the length values so that the mean diameter matched the mean length.

### 4.5 Statistics

All statistical tests were performed with Matlab. Kruskal–Wallis tests with a 5% significance level were used to evaluate the statistical differences among multiple groups (Fig. 2).

For the random assignment control (Fig. S4 in the Supplementary Material), we shuffled all spines to a random dendrite, keeping the number of spines on every dendrite constant.

We used Pearson’s chi-square test (Matlab’s function crosstab) to test for significant differences in class distribution on dendrites (Fig. 2) with a 5% significance level and corrected for multiple comparisons with the Bonferroni correction.

## Supplementary Material



## Data Availability

The code and data presented in this study are available upon reasonable request to the corresponding author.
