# Supplementary material for: Distribution of spine classes shows intra-neuronal dendritic heterogeneity in mouse cortex
Source: Neurophotonics. 2024 Dec 19;12(1):015001. doi: 10.1117/1.NPh.12.1.015001 (PMC11657875; doi:10.1117/1.NPh.12.1.015001)
Supplement: Supplementary file 1 [file NPh_012_015001_SD001.pdf]

Supplementary Material

to

Distribution of spine classes shows intra-neuronal dendritic heterogeneity in mouse cortex

Carina C. Theobald, Ahmadali Lotfinia, Jan A. Knobloch, Yasser Medlej, David Stevens, Marcel A. Lauterbach

Saarland University, Molecular Imaging, Center for Integrative Physiology and Molecular Medicine, Homburg, Germany

Figure S1 Spine classes along dendrites

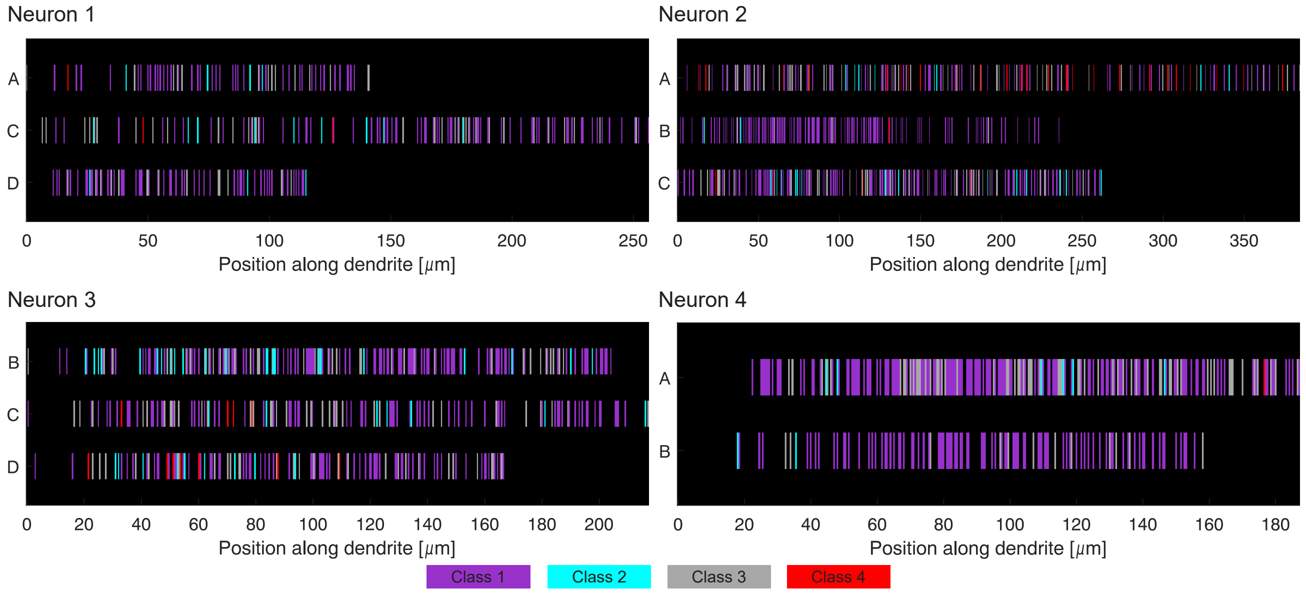

Spine classes along dendrites Individual spines are represented by lines with the color code of the classes. Each row represents one dendrite.

Figure S2 Spines classes are inhomogeneously distributed on dendrites that were fixed before slicing

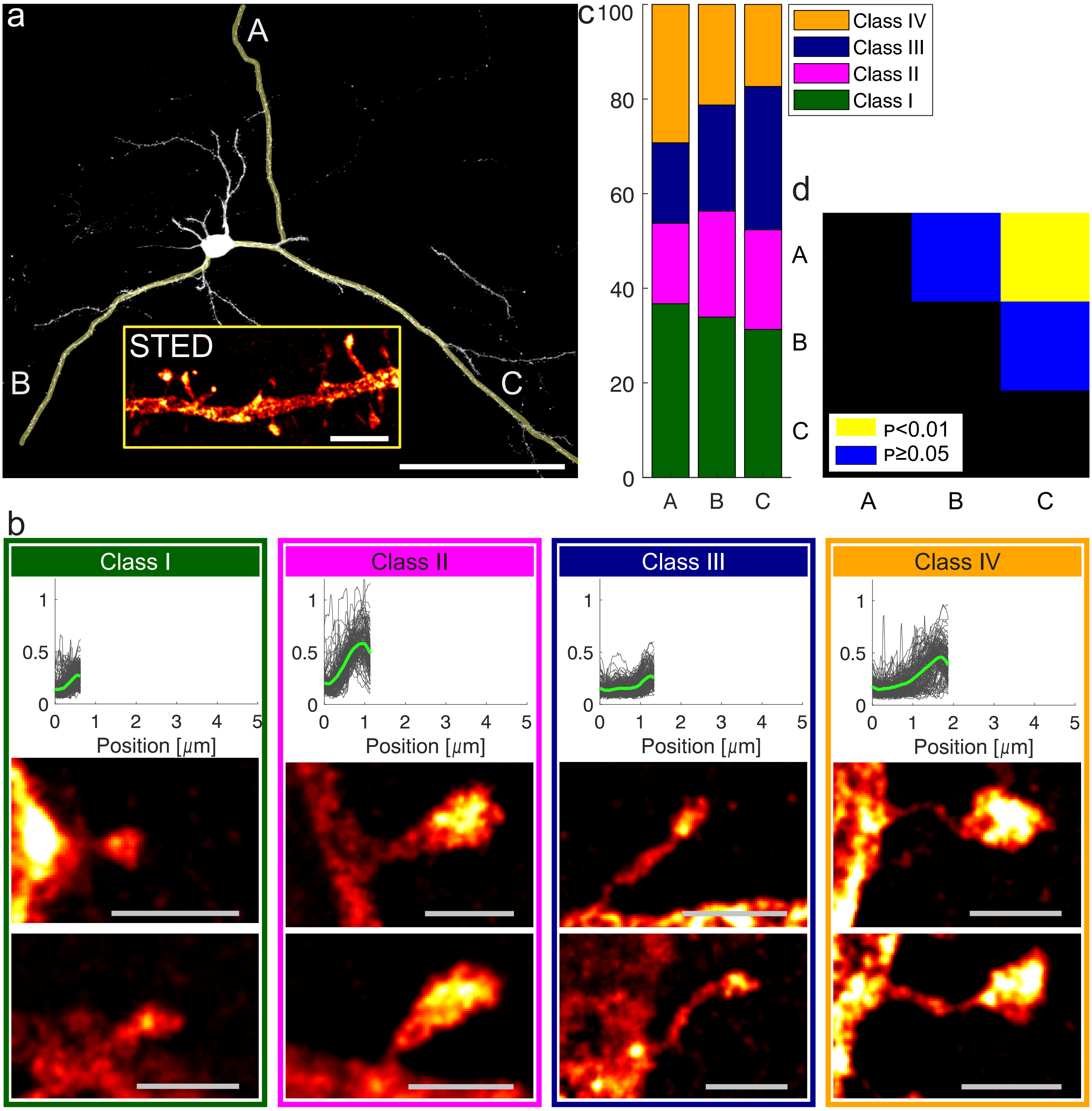

Spines classes are inhomogeneously distributed on dendrites that were fixed before slicing (a) Confocal overview of neuron expressing tdTomato (maximum-intensity projection). Letters and yellow markings identify individual dendrites, which were then imaged with STED microscopy (inset). Scale bar 50 µm, 5 µm in the inset. (b) Spines were categorized by hierarchical clustering of shape and length, using only the spines of the tdTomato-marked neuron, not mixing them with the other staining method (note that very few filopodia-like spines were present here, they form thus not an own class). Upper panels: Individual diameter profiles (gray) and average profile of each class (green). Total length of the profiles were scaled to the average spine length of the respective class. Lower panels: representative spine examples. Scale bars 1 µm. (c) Relative abundance of each spine class on the dendrites (A–C as marked in panel a), the distribution differs between dendrites. (d) Pairwise Person's chi-square tests confirm significant differences between the repartition of the classes on the dendrites A and C. p values color coded: $p \leq 0.01$ yellow, $p > 0.05$ blue. All tests have been corrected for multiple comparisons. Refer to supplementary methods for further details.

Figure S3 Pairwise dependences of spine parameters and relationship with spine class

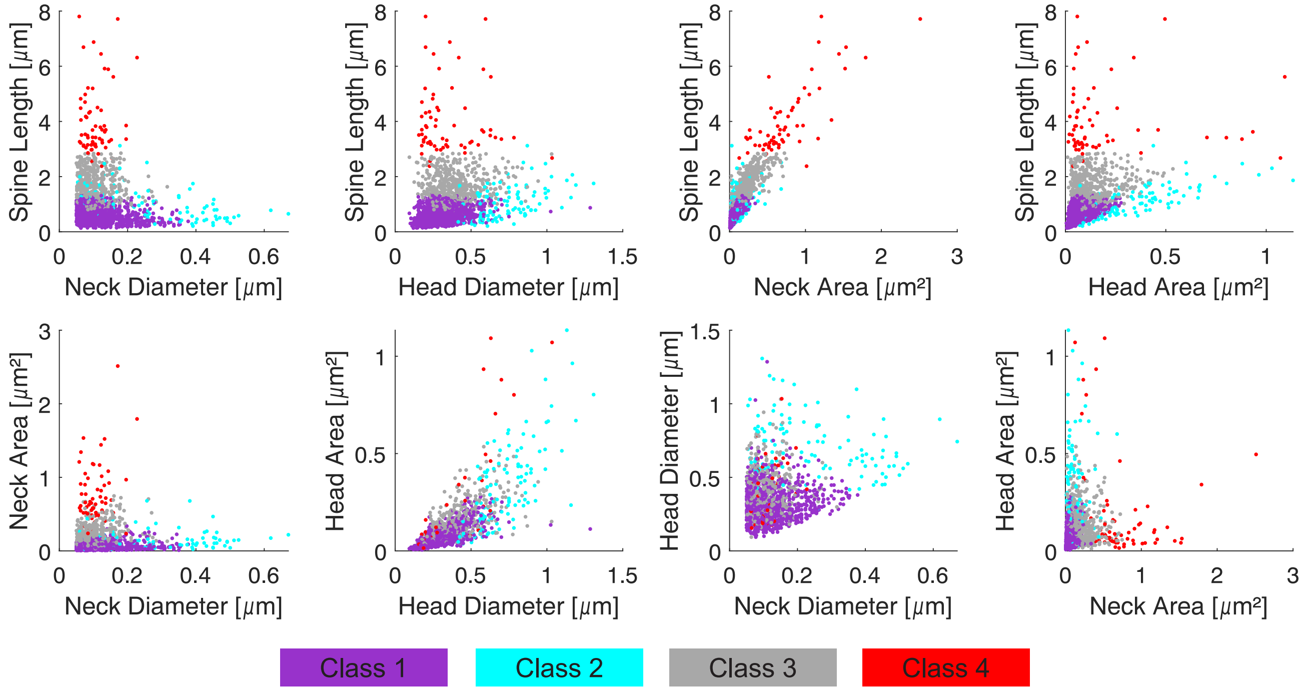

Pairwise dependences of spine parameters and relationship with spine class Each dot represents one spine; colors denote spine class with color code as in Fig. 2.

Figure S4 Control: Spine paramters are not distinct between dendrites if spines are assigned randomly

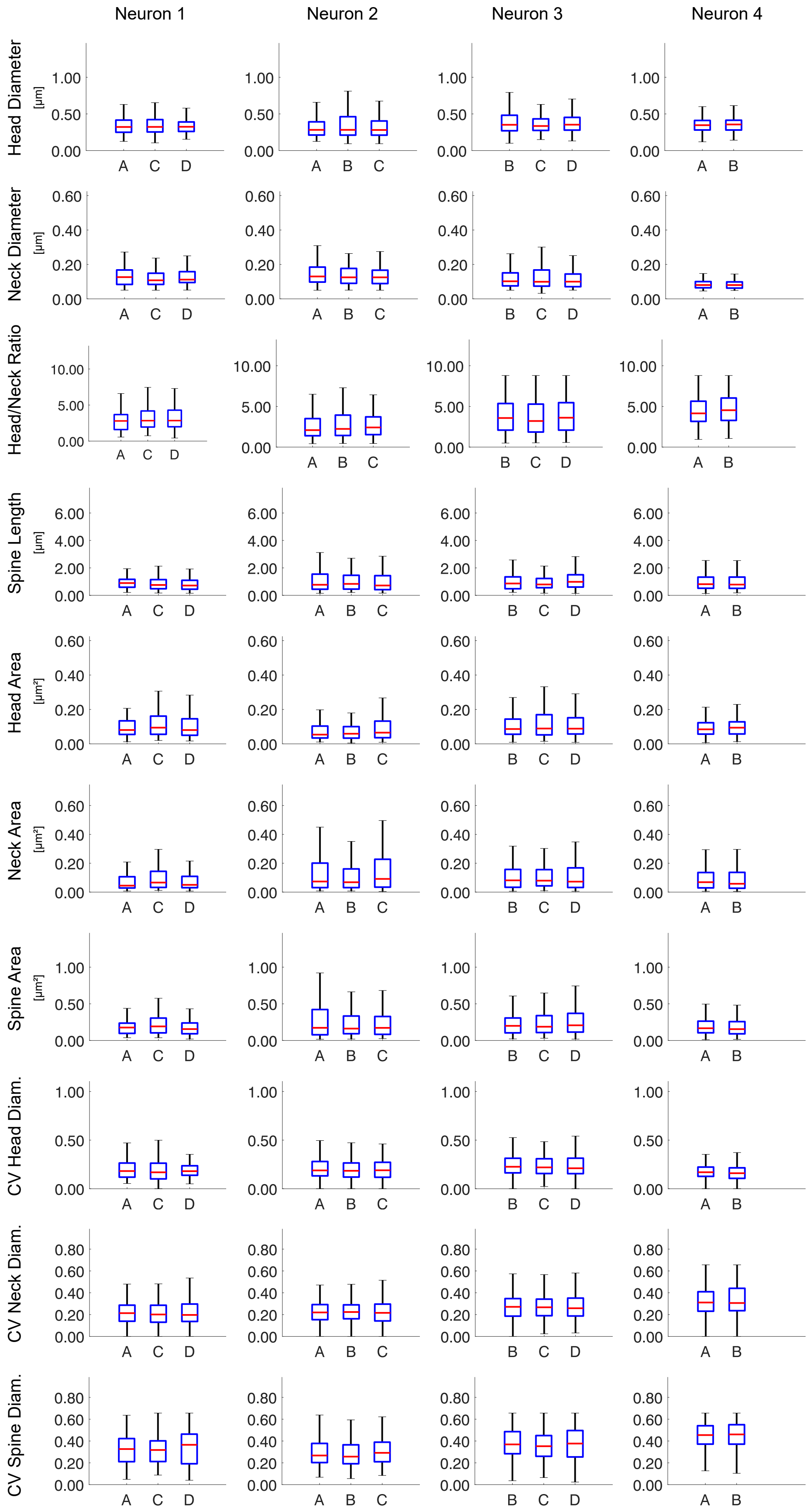

Control: Spine paramters are not distinct between dendrites if spines are assigned randomly When spines were randomly reassigned to different dendrites of a particular neuron, the significant differences in spine descriptors between the dendrites disappeared.

Figure S5 Spinules decorate some spine classes more often than others

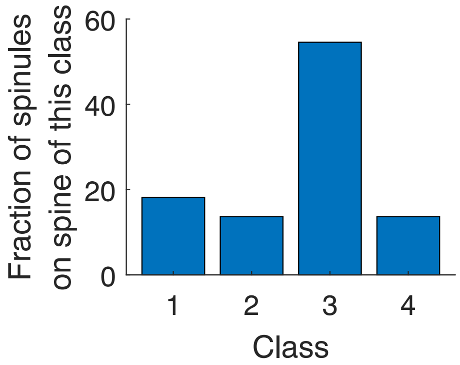

Spinules decorate some spine classes more often than others Calculating the fraction of spinules that decorate spines of a certain class shows that spinules decorate spines of class 3 more often than spines of the other classes.

Figure S6 Spinules in tdTomato marked neurons

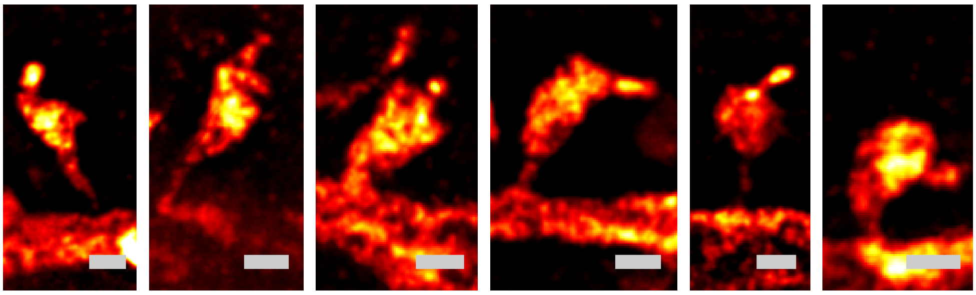

Spinules in tdTomato marked neurons On the neurons that express the fluorescent marker protein tdTomato various forms of spinules are observed as well. Scale bars 500 nm.

Supplementary methods: sample preparation and analysis of genetically marked neuron (Fig. S2)

To obtain sparse neuronal labeling, we profited from off-target labeling of neurons in TgN(GFAP-CreERT2) mice [49] crossed with the reporter line TgH(ROSA26-CAG-lsl-tdTomato-WPRE)^{Ai14} [50].

Adult female mice were intraperitoneally injected with a single low dose (25 mg/kg) of Tamoxifen (Carbolution, Neunkirchen, Germany), dissolved in Miglyol 812 (Caesar & Lorentz, Hilden, Germany) and transcardially perfused with 4% paraformaldehyde (PFA) one week later. After overnight postfixation in PFA (4% at 4 °C) 35-µm-thick free floating sagittal vibratome slices were incubated for one hour with blocking buffer (5% horse serum with 0.5% Triton X100 in PBS) and then incubated overnight at 4 °C with primary antibody against DsRed (Rabbit polyclonal antibody 632496, Clontech/TaKaRa, dilution 1:1000). Slices were washed three times for 10 min in PBS and then incubated with secondary goat anti-rabbit Atto647N antibody (Sigma, dilution 1:200) for 2 h at room temperature. After three final washing steps in PBS, slices were mounted in Mowiol on # 1.5H coverslips (Marienfeld, Lauda-Königshofen, Germany).

Imaging was performed as described in the main methods. Power of the STED laser was set to about 270 mW in the back focal plane of the objective. A cortical neuron was chosen close to the slice surface with three dendrites in a depth below 20 µm.

Clustering of spines was performed as described in the main methods. To avoid clustering spines that have been stained with different methods, the clustering was performed independently of the neurons that were stained via patching (Figs 1 and 2); only the tdTomato stained spines were included. The cutoff was set to have four clusters (denoted I–IV) again.
